# Epigenetics enters the stage in vascular malformations

**DOI:** 10.1172/JCI182904

**Published:** 2024-08-01

**Authors:** Salim Abdelilah-Seyfried

**Affiliations:** Institute of Biochemistry and Biology, Potsdam University, Potsdam, Germany.

## Abstract

Cerebral arteriovenous malformations represent the most common form of vascular malformations and can cause recurrent bleeding and hemorrhagic stroke. The current issue of the *JCI* features an article by Zhao et al. describing a mouse model of cerebral arteriovenous malformations. Endothelial cells lacking matrix Gla protein, a BMP inhibitor, underwent epigenetic changes characteristic of an endothelial-to-mesenchymal fate transition. The authors uncovered a two-step process for this transition controlled by the epigenetic regulator histone deacetylase 2 (HDAC2), which controls endothelial cell differentiation, and by enhancer of zeste homolog 1 (EZH1), which suppressed mesenchymal fate. This discovery provides a promising entry point for preventive pharmacological interventions.

## A need for animal models of AVMs

Vascular malformations (VMs) are genetic diseases of the blood or lymphatic endothelium. They include cerebral arteriovenous malformations (AVMs) characterized by thickened blood vessels bypassing capillaries, which connect brain arteries and veins. Affected blood vessels are prone to rupture, with devastating consequences for patients. Currently, there are no preventive treatment options available. Hence, there is an urgent need for animal models that enable researchers to characterize the molecular underpinnings of cerebral AVMs and offer hope of discovering preventive interventions.

Genetic and molecular studies have led to the discovery that the development of some AVMs can be linked to defective BMP signaling. This is the case in the rare vascular disease hereditary hemorrhagic telangiectasia (HHT). Patients with HHT harbor loss-of-function mutations in genes encoding activin receptor-like kinase 1 (ALK1) (1), its coreceptor ENDOGLIN (2), or the downstream transcription factor SMAD4 (3). Consequently, AVMs can occur in many organs. This issue of the *JCI* features an article by Zhao et al. describing a mouse model of cerebral AVMs based on the endothelial-specific knockout of the BMP inhibitor matrix Gla protein (MGP) (4).

Previous research had shown that MGP levels were reduced in ALK1-deficient mice and loss of MGP caused AVMs in different organs and the brain (5, 6). Strikingly, the activation of MGP suppressed the formation of AVMs in ALK1-deficient animals, providing evidence for a role of MGP in the etiopathology of AVMs in HHT (7). Patients with a loss of *MGP* present with the rare disease Keutel syndrome, which involves cardiovascular defects (reviewed in ref. 8).

## Epigenetic changes in AVMs

There was strong evidence that BMP signaling is involved in AVM formation upon loss of MGP. Yet the precise mechanism of disease progression has remained mostly unknown. Zhao et al. (4) discovered changes to the cell and molecular biology of affected endothelial cells in murine endothelial-specific *Mgp*-knockout models with cerebral AVMs and in human brain microvascular endothelial cells with a CRISPR/Cas9 knockout of *MGP*. Their work revealed that endothelial cells undergo a shift from an endothelial cell fate, as indicated by reduced expression of the endothelial marker genes *Kdr* and *vWF*, toward a mesenchymal fate, as indicated by the upregulation of markers including *Snail1*.

The authors exploited this observation to develop an assay based on the expression of the *Snail1* promoter driving GFP in MGP CRISPR cells. This cell line was used to initiate a pharmacological suppression screen using compound libraries. Unexpectedly, HC toxin, an inhibitor of histone deacetylase 2 (HDAC2), reduced the expression of GFP from the *Snail1* promoter and alleviated AVM formation in the murine endothelial-specific *Mgp*-knockout model. This finding strongly hinted at an involvement of epigenetic changes mediated by HDAC2 in the etiopathology of brain AVMs.

Changes from an epithelial or endothelial to a mesenchymal cell fate are a hallmark of many developmental processes or cancer. In fact, there are many well-studied examples of such fate transitions that are controlled by epigenetic regulation (9). However, Zhao and authors described the contribution of epigenetic regulation to pathological endothelial-to-mesenchymal transition (EndMT) processes as being associated with VMs (4).

## Shifting endothelial toward mesenchymal cell fates in AVMs

A number of epigenetic regulatory proteins, including HDAC2 or components of the polycomb repressive complexes, such as enhancer of zeste homolog 1 (EZH1), catalyze modifications on histone residues, affecting the state of the chromatin network. In turn, this can alter the accessibility of gene loci for transcriptional activation. Specifically, HDAC2 removes acetylation marks on histone lysine residues (H4K8ac), which are activating marks (10). Consequently, HDAC2 activity results in the transcriptional silencing of genes. Similarly, EZH1 catalyzes histone trimethylation (H3K27me3), which has an inhibitory effect on gene transcription (11–13). When systematically screening for expression changes of epigenetic regulatory proteins in MGP-depleted murine brain endothelial cells, HDAC2 was strongly upregulated while EZH1 was downregulated. But what was the relationship between these two proteins and how did this affect gene expression?

**Figure 1:**
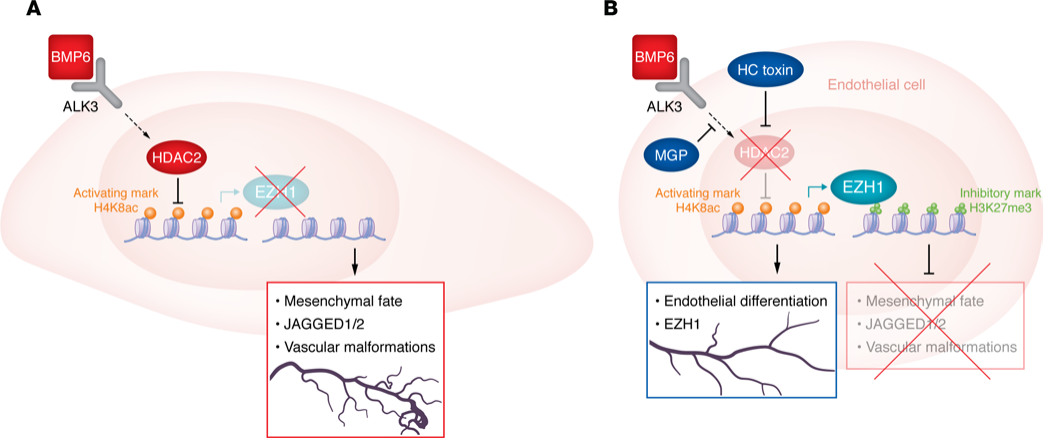
Epigenetic changes causing an EndMT in cerebral AVMs reveal a targetable pathway. (**A**) The activation of mesenchymal genes and JAGGED1/2 has been implicated in the formation of cerebral AVMs. Strong activation of HDAC2 results in transition from the endothelial cell state to a mesenchymal fate (EndMT) and activation of JAGGED1/2. (**B**) In healthy conditions, EZH1 suppresses the activation of mesenchymal genes and JAGGED1/2. MGP activity suppresses signaling by BMP6/ALK3, preventing the activation of HDAC2. This suppression ensures a two-tier molecular regulatory mechanism in preventing EndMT and cerebral AVMs: (i) suppression of HDAC2 alleviates the negative control of genes involved in endothelial differentiation and of *Ezh1*; (ii) EZH1 methylates genes associated with mesenchymal fate, thus making them less accessible. These pathways offer an entry point for preventive pharmacological interventions in cerebral AVMs that are characterized by a strong activation of HDAC2. Zhao et al. demonstrate an effective treatment for cerebral AVMs using the HDAC2 inhibitor HC toxin in a mouse model.

These questions were answered when the authors found that the depletion of HDAC2 in human MGP CRISPR cells normalized the expression of endothelial differentiation genes such as *KDR* and restored the expression of *EZH1*. Similarly, the depletion of HDAC2 also abolished the expression of many mesenchymal marker genes. However, overexpression of EZH1 in human MGP CRISPR cells using a CMV promoter–driven construct only suppressed mesenchymal gene expression, but had no effect on endothelial differentiation genes, which were still suppressed. These findings established a two-tier molecular regulatory mechanism in the control of EndMT in cerebral AVMs whereby (a) HDAC2 negatively controls the expression of endothelial differentiation genes and EZH1 and (b) EZH1 suppresses the activation of mesenchymal genes. Hence, low levels of EZH1 promote mesenchymal cell fates (Figure 1).

However, these findings left unresolved the question of how the loss of MGP and altered BMP signaling caused an elevated expression of HDAC2. To address this question, the authors treated human brain microvascular endothelial cells with different BMP ligands. BMP6 caused an induction of HDAC2, and this effect involved the BMP type I receptor ALK3. This finding suggested that the BMP inhibitor MGP suppresses BMP6/ALK3-induced HDAC2 activity, thereby securing endothelial cell differentiation and suppressing mesenchymal fates.

## Conclusions

This work by Zhao and authors presents an exciting discovery in the field of VMs; it links epigenetic changes in the genome with pathological changes of endothelial cells, shifting them toward a mesenchymal fate (4). The process of pathological EndMT is not restricted to cerebral AVMs, but has also been described in other VM diseases, including cerebral cavernous malformations (14). It will be an exciting endeavor for future research to explore whether there is a derailed epigenetic regulation that can be targeted by pharmacological intervention and that will come to benefit patients with cerebral AVMs and other pathologies of the vasculature.

## Version 1 08/01/2024

Electronic publication
